# Exploring Interaction Dynamics in Dog-Assisted Therapy: An Observational Study

**DOI:** 10.3390/bs15081115

**Published:** 2025-08-18

**Authors:** Candela Jasmin Hüsgen, Nienke Peters-Scheffer, Robert Didden

**Affiliations:** 1 Behavioural Science Institute, Radboud University, P.O. Box 9104, 6500 HE Nijmegen, The Netherlands; nienke.peters-scheffer@ru.nl (N.P.-S.); robert.didden@ru.nl (R.D.); 2 Hulphond, P.O. Box 24, 5373 ZG Herpen, The Netherlands; 3 Trajectum, Dokter Stolteweg 17, 8025 AV Zwolle, The Netherlands

**Keywords:** dog-assisted therapy (DAT), animal assisted therapy, human–animal interaction, behavioural observation, dog behaviour

## Abstract

(1) Background: Dog-assisted therapy (DAT) integrates dogs into therapeutic sessions to enhance participants’ physical, emotional, and social well-being. Despite its growing popularity, little is known about the interaction dynamics between the dog, participant, and therapist during sessions. (2) Methods: This study examined these dynamics, focusing on active participation, focus direction, joint focus, and physical contact. Video data from sessions 1, 5, and 9 of 10 individual therapy sessions with five participants were analysed using behavioural observations and an ethogram. (3) Results: Results indicated that therapists’ active participation increased over time while participants’ activity levels remained stable. Dogs were most active during the initial and final sessions. Participants’ focus on therapists remained consistent, but their focus on the dog stabilised after an initial decline. Dogs are primarily focused on their surroundings. The joint focus between participants and therapists increased, and physical contact with dogs varied significantly among participants and dogs. (4) Conclusions: The findings partially support the “icebreaker” theory, whereby dogs help establish initial rapport. However, the trend was not consistent across all participants. Therapist–dog interactions remained low and stable. Differences in dog characteristics (e.g., breed and fur type) and participant needs may explain variation in physical contact. These findings underline the complexity of DAT and highlight the need for further research into interaction patterns relate to participants and dog characteristics.

## 1. Introduction

Although research on dog-assisted therapy (DAT) has promising outcomes, little is known about the interactions between dogs, therapists, and children within DAT and how these interactions relate to the context and goals of the intervention (Binfet et al., 2021; Jones et al., 2019). Current studies suggest DATs potentially enhance participants’ physical, emotional, and social well-being (Grové et al., 2021; Guzmán et al., 2022; Lavín-Pérez et al., 2021). Research highlights that the presence of dogs during therapy can reduce anxiety, elevate mood, and foster social interactions, positioning DAT as a valuable supplement to traditional therapeutic approaches (Grajfoner et al., 2017; Van Schooten et al., 2024; Wu & Wei, 2023). However, existing studies focused on outcomes, often overlooking how the interactions between the child, dog and therapist drive these results (Hüsgen et al., 2022; Wagner et al., 2022). Understanding these interactions is crucial for explaining DAT’s effectiveness and optimising therapeutic outcomes (De Santis et al., 2024).

In human–animal interactions, the triadic relationship between the dog, the participant, and the therapist provides the setting in which the participant is learning. The triadic relationship between the therapist, participant, and dog distinguishes DAT from many other therapies, which typically involve a dyadic relationship between the participant and therapist. Observable interactions, such as movements, focus, and physical contact, are presumably distributed differently in this triadic dynamic, creating a therapeutic setting with both advantages and challenges (Glenk & Foltin, 2021; IAHAIO, 2018; Roma et al., 2021; ISAAT, 2024; Voutilainen & Peräkylä, 2016). A challenge, for example, is that according to the One Health concept, the therapist in Dog-Assisted Therapy (DAT) holds responsibility not only for the client’s well-being but also for their own welfare and that of the dog (IAHAIO, 2018). The pre-established relationship between the therapist and the dog plays a crucial role in modelling a secure and trusting bond for the participant, facilitating the therapeutic alliance (A.MOET A. ERBIJ? Fine, 2018). It can be an advantage of the setting. Unlike other therapeutic alliances, the therapist and dog enter this process with a relationship built before the therapy session, outside the therapeutic context. This relationship, grounded in training and mutual understanding, includes the therapist’s responsibility for the dog’s actions and welfare (ISAAT, 2024; IDEM). While the therapist–participant relationship is developed during the session, the therapist–dog relationship serves as a model of safety and trust for the participant.

Observations can be used to analyse interactions in therapy by recording actions such as movement, physical contact, and the direction of participants’ focus (Binfet et al., 2021; Ramseyer & Tschacher, 2011). DAT often involves active engagement, including movement and physical contact, particularly in sessions encouraging interaction between the dog and the participant (Abadi et al., 2022; Wohlfarth et al., 2013). Examples include agility exercises, obedience training, or simply taking a walk together. These movements are comparable to those in psychomotor therapy, where integrating movement into therapy can support emotional processing, enhance emotional resilience, aid motor function recovery, and foster creativity and exploration (Bergland et al., 2018). This approach encourages experiential learning through shared reflection on the experiences. Another interaction in DAT is petting the dog, which introduces physical contact (Beetz et al., 2012). Studies suggest that such actions may influence participants’ emotional states by fostering attachment and a sense of safety, potentially enhancing openness to therapy. Despite these insights, little is known about the specific frequencies of these interactions during sessions, which this study aims to address.

This study aims to determine the extent to which observable interactions in DAT, such as movements, focus, joint focus, and physical contact, occur during therapy sessions. Physical contact has been shown to provide therapeutic benefits. Similarly, moments of joint focus between the participant, therapist, and dog create opportunities for engagement and interaction within the triadic relationship. These interactions are believed to enhance the therapeutic process. To better understand these dynamics and their contribution to the effectiveness of DAT, this study seeks to answer the following research question: What are the frequencies of movement, physical contact, and focus in DAT sessions, and can patterns of these interactions be identified?

## 2. Materials and Methods

### 2.1. Participants and Setting

Participant recruitment was conducted through social media, a local child and family service centre, and participant-to-participant conversations. The inclusion criteria were as follows: (1) children aged 10–18 years; (2) a treatment goal related to social-emotional challenges, focus, and/or impulse control; and (3) the ability to attend therapy once a week. Exclusion criteria included (1) allergies to dogs, (2) aggression towards humans or animals, and (3) a fear of dogs. In total, five adolescents participated as follows: two boys aged 12 and 15 years, and three girls aged 13, 18, and 18. The participants were coping with challenges such as emotional regulation, Autism Spectrum Disorder (ASD), performance anxiety, perfectionism, distractibility, and Attention-Deficit/Hyperactivity Disorder (ADHD). Only the 15-year-old boy had a dog at home. Each participant attended ten weekly sessions over a 12-week period. Four therapists provided the interventions. All had completed in-company Dog-Assisted Therapy (DAT) training at Hulphond Nederland, a foundation specialised in training assistance dogs and providing a wide range of animal-assisted interventions in the Netherlands. The therapists had between one and three years of experience in delivering DAT. Three dogs participated in the study—a black male Labrador (8 years old), a white male Poodle (5 years old), and a blond male Golden Retriever (4 years old). All dogs began their training at approximately eight weeks of age and started working in therapy between 20 and 24 months. They worked two days per week in therapeutic settings and had each been active in DAT for at least one year. Each dog was paired with a specific therapist, and the dog–therapist teams had developed a working relationship before the study’s start, as they had worked together for at least a year, ensuring consistency and trust within the sessions. Additional dogs and therapists had been at the research location several times before the research began to become familiar with the room, smells, and overall environment.

### 2.2. Therapy Sessions

Therapy sessions were held in a controlled environment conducive to therapy and dog interactions at Radboud University’s Baby and Child Laboratory. The guidelines outlined in the IAHAIO whitepaper for integrating dogs into therapy settings were followed during all sessions. Ethical considerations were paramount in the study. Informed consent was obtained from all participants and their legal guardians, and the anonymity and confidentiality of the participants were maintained throughout the study. The institutional ethics committee review board of the Radboud University approved the study (ECSW-2022-022), adhering to ethical guidelines for research involving minors and animals.

**Figure 1:**
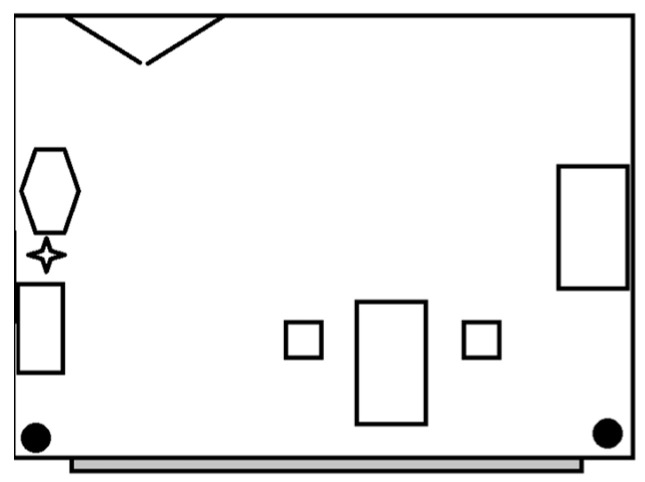
Floor plan of the therapy room. Including double doors, two tables, two chairs, a storage cabinet, a dog bed (hexagon), a dog water bowl (star), and two cameras (black circles).

Video footage was systematically recorded using high-definition cameras from HikVision (DS-2CD2183G2-I), placed unobtrusively in the therapy room to ensure that all interactions were captured without disrupting the therapy sessions. Two cameras were placed in the two corners of the room at a height of 2.3 m to view the participants from multiple angles. The therapy room measures 5 by 7 m (see Figure 1). On one side of the room, double doors provide access. A large window directly opposite allows natural light to enter. The floor is covered with carpet, which offers grip for the dog. Additionally, a resting area for the dog was included with a dog bed and a water bowl. The room is furnished with two tables (each measuring approximately 140 × 80 cm) and two chairs, as well as a storage cabinet (100 × 50 cm). The two cameras are installed in the top-right and bottom-right corners of the room. During sessions, the therapist, client, and dog were in the room.

A researcher who observed the video recordings in real time adjusted the cameras during the sessions as necessary. Additionally, the therapy room contained two tables and four chairs, a cupboard with dog toys, agility materials such as a tunnel and cones, and treats for the dog.

Each therapy session followed a consistent structure, beginning with an introduction or welcome, during which participants discussed their daily life issues and feelings. This was followed by a warm-up exercise, which remained the same across all sessions, helping the participant to establish familiarity with the therapist and the dog. For example, the warming-up could be a small sequence in which the client gives the dog simple commands such as “sit”, “down”, and “shake hands” to initiate contact and build engagement. After the warm-up exercise, the participant engaged in an individualised exercise tailored to their therapeutic goals, such as a personalised obstacle course, adapted to the client’s therapeutic goals and the dog’s abilities. This was followed by a reflective conversation with the therapist and the participant to encourage personal insights and reinforce learning. Next, a second exercise was introduced. Sometimes, this exercise built upon the previous exercise to deepen engagement; in other sessions, a new exercise was introduced. This second activity followed a reflective conversation, allowing participants to process and internalise their experiences. Each session concluded with a dog puzzle activity, in which the dog was given a puzzle task. This final exercise provided a light-hearted end to the session. It allowed participants to observe the dog’s problem-solving abilities, creating a positive and relaxed atmosphere as the session unfolded.

### 2.3. Measures

The entire sessions, each lasting between 40 and 60 min, were video recorded and used for analysis. Recordings from the first, fifth, and ninth sessions of each participant were included in full and divided into 30 s segments. Each session was scored with an ethogram developed for this study. Momentary time sampling was employed to record behaviour at specific predetermined moments (Harrop & Daniels, 1986). This approach ensures a systematic and consistent method for recording and analysing the interactions within the sessions. The ethogram comprised 25 categories, divided into ten for the dog, eight for the participant, and eight for the therapist (Appendix A). The ethogram was based on the literature and the principles of Behavioural Observation (Ramachandran, 2012) commonly applied in psychotherapy evaluations. Table 1 describes the observed behaviours and interactions categorised into movements, focus direction, joint focus and physical contact. The category of movements is divided into passive and active actions, distinguishing between stationary behaviours, such as sitting, standing, or lying down, and dynamic behaviours, such as walking.

### 2.4. Data Processing and Analysis

First, the data were standardised by adjusting the frequency of observations based on session length, ensuring comparability across all sessions despite differences in duration. Initially, the active and passive behaviours of the dog, participant, and therapist were analysed. First, the active and passive behaviour scores were calculated by recording movement observations into two categories—active and passive. Behaviours are classified as active, walking and moving, or passive, such as sitting, standing, or lying down. Then, cross-tabulations were used to identify patterns and frequencies. This provided an overview of how much time each participant spent engaging in active versus passive behaviours during the therapy sessions. Second, the focus direction of each participant was examined, categorising where the focus was directed—whether toward the participant, the therapist, the dog, or something else. This analysis helped reveal shifts in focus and moments of joint focus. Subsequently, the joint focus was analysed to capture instances where two participants were simultaneously focused. Physical contact between the dog and the participant or therapist was recorded, capturing all forms of touch, including petting and other types of physical interaction.

## 3. Results

### 3.1. Frequencies of Active and Passive Participation During Therapy Sessions

**Figure 2:**
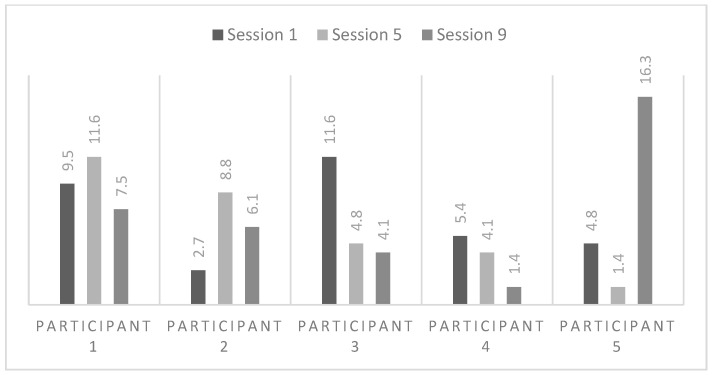
Participants’ active participation across three therapy sessions is displayed in percentage.

The participation levels of the dogs fluctuated across the sessions as follows: 37.9% of the intervals in Session 1, 21.4% of the intervals in Session 5, and 40.7% of the intervals in Session 9, where the dog was active. In contrast, participant participation remained relatively stable (34% in Session 1, 30.6% in Session 5, and 35.4% in Session 9). Therapists demonstrated increasing participation rates from 28.1% in Session 1 to 33.7% in Session 5 and 38.2% in Session 9. The level of participant participation was consistent across all sessions, reflecting the structured nature of the therapy. Therapists started with a low level of active engagement in Session 1 (28.1%), which gradually increased in subsequent sessions. Dogs exhibited more dynamic levels of participation, with a noticeable decline in Session 5, followed by an increase in Session 9. The trend for participant participation remained stable across all sessions, indicating consistent involvement in the therapy process. Therapists displayed an upward trend in participation over time, suggesting increasing activity as sessions progressed. The trend for dogs was more irregular, with no clear directional pattern, highlighting fluctuations in their engagement throughout the sessions. Figure 2 provides a detailed view of individual participant trends. Participant 3 exhibited the highest initial participation but steadily declined over the sessions. In contrast, Participant 5 demonstrated an upward trend, with significant growth in active engagement. The remaining participants displayed stable patterns or slight fluctuations in their participation levels over time.

### 3.2. Frequencies of Focus Direction During Therapy Sessions

Table 1 displays therapists’ focus direction during sessions involving participants and dogs, categorised into three areas as follows: focus direction towards the participant, towards the dog, and others. Data from the three sessions and average values for each session are presented in Table 1. In terms of focus direction from the therapist towards the participant, therapists showed an increase from Session 1 (53%) to Session 5 (64%), followed by a slight decrease in Session 9 (57%). The focus of the therapist on the dog was initially high in Session 1 (30%), dropped in Session 5 (20%), and rose again in Session 9 (27%). Focus directed towards other aspects stayed stable from Session 1 (18%) to Session 5 (16%) and in Session 9 (16%). While a pattern emerged in the therapist’s focus direction toward the participant, and no consistent pattern was found in the therapist’s focus on the dog. Therapists demonstrated the highest focus on the participant during Session 2, whereas their focus on the dog varied, with noticeable differences between individual therapists.

Table 2 provides insights into participants’ focus direction during therapy sessions, categorised into the following three areas: towards the therapist, towards the dog, and others. Data from Session 1, Session 5, and Session 9 for five participants and average values are presented. The average focus on the therapist stayed stable from 44% in Session 1 to 47% in Session 5, and then to 45% in Session 9. Notable individual variation is observed, with some participants increasing their focus on the therapist. Initially, participants’ focus on the dog was 37% in Session 1, dropping to 26% in Session 5 and then rising again to 33% in Session 9. Participants’ focus on other factors increased from 20% in Session 1 to 27% in Session 5, then decreased slightly to 23% in Session 9.

Table 3 presents data on dogs’ focus direction during therapy sessions, categorised into three areas as follows: towards the therapist, towards the participant, and others. Data from three sessions for five participants are provided, along with average values. Regarding the focus on the therapist, the dogs showed an average of 12% in Session 1, which slightly decreased to 10% in Session 5 and further decreased to 6% in Session 9. When focusing on the participant, the dogs’ focus direction was 25% in Session 1, increased to 28% in Session 5, and slightly decreased to 24% in Session 9. The dog’s most measured focus direction was categorised as “other”. The average focus on other factors was 63% in Session 1, stayed stable at 62% in Session 5, but then rose to 71% in Session 9. The data indicate that dogs in therapy sessions have a fluctuating focus direction span, with the most consistent focus being on factors other than the therapist or participant. Their focus direction towards the therapist decreased over time, while their focus on the participant showed some variability.

### 3.3. Frequencies of Joint Focus During Therapy Sessions

Based on the focus direction coding, the proportion of time during which joint focus occurred was calculated, as shown in Table 4. Joint focus was defined as moments when two of the three individuals involved simultaneously directed their attention toward each other. For each session, the percentage of joint focus was computed for the following three dyads: participant–dog, therapist–dog, and participant–therapist. The results indicate that joint focus between the participant and the dog decreases over time, from an average of 18% in session 1 to 15% in session 9. Notably, individual variation is considerable as follows: some participants maintain a relatively high level of shared attention with the dog in session 5, whereas for others, this declines more rapidly. Joint focus between therapist and dog remains consistently low across all three sessions, averaging 3% in session 1 and approximately 2% in sessions 5 and 9. In contrast, joint focus between participant and therapist increases over time. In session 1, an average of 34% of the session involved joint focus, which rises to 43.1% in session 5 and remains at 41.3% in session 9.

### 3.4. Frequencies of Physical Contact During DAT Sessions

**Figure 3:**
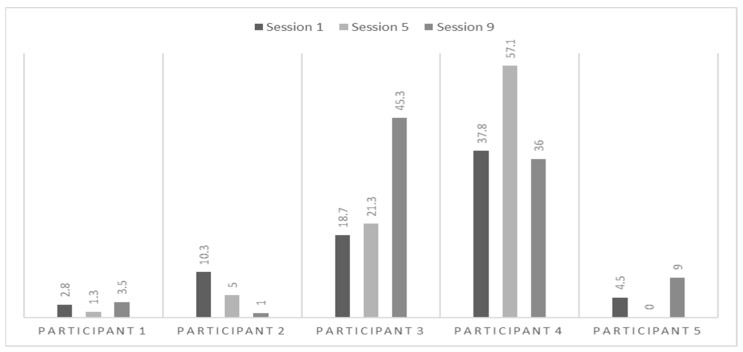
Percentage of time participants spent petting their dogs during therapy sessions.

Figure 3 illustrates the percentage of time each participant spent petting the dog during the three therapy sessions. It shows the varying levels of physical contact between the participants and their dogs. Participant 1 displayed consistent behaviour across the sessions, spending 2.8%, 1.3%, and 3.5% of the time in contact with his dog during Sessions 1, 5, and 9. This indicates relatively low and stable physical contact. In contrast, Participant 2, a boy interacting with a Labrador, showed a peak of physical contact during Session 1, where 10.3% of the session was spent petting the dog. However, this interaction dropped to 5% in Session 5 and 1% in Session 9, showing decreased physical engagement. On the other hand, Participant 3, a 18-year-old girl interacting with a Poodle, demonstrated increasing engagement across the sessions. She began with 18.7% in Session 1, increased to 21.3% in Session 5, and saw a substantial rise to 45.3% in Session 9. Similarly, Participant 4, an 18-year-old girl interacting with a Poodle, displayed dynamic interaction patterns. She spent 37.8% of the time petting her dog in Session 1, which increased to 57.1% in Session 5 before slightly dropping to 36% in Session 9. These two participants, who worked with Poodles that also tend to seek physical contact on their own, displayed the highest levels of physical engagement, likely influenced by the dogs’ preference for this type of interaction. Lastly, Participant 5, a 13-year-old girl interacting with a Labrador, showed minimal engagement, petting her dog 4.5% of the time in Session 1. Interestingly, she had no recorded physical contact in Session 5 but increased to 9% in Session 9, indicating some variability in her interaction levels. The analysis of physical contact between participants and their dogs reveals distinct trends. Participants interacting with Poodles (P3 and P4) showed the highest and increasing levels of physical contact over time. In contrast, Participants 1 and 5, interacting with a Golden Retriever and a Labrador, exhibited consistently low and stable physical contact throughout the sessions. Participant 2, also with a Labrador, initially had higher contact but showed a notable decrease over time.

## 4. Discussion

This study explored the interactions within DAT sessions by examining active and passive participation, focus direction, joint focus, and physical contact among the dog, participant, and therapist. Analysis of their involvement during the therapy sessions showed that participants remained actively involved at similar levels across all sessions, while therapists exhibited a slight increase in activity over time. The dogs showed the most variation in participation, with their activity levels fluctuating throughout the sessions, being more active at sessions one and nine than during Session 5. Further analysis showed that therapists primarily focused on the participants, and participants’ focus on the therapists increased slightly across the sessions. At the same time, participants’ focus towards the dogs decreased. However, this trend was not consistent across all participants. Joint focus analyses revealed that engagement between participants and dogs slightly decreased, while joint focus between participants and therapists increased in most cases. Physical contact between the participant and the dog varied significantly, with the Poodle exhibiting considerably more physical contact with participants than the other dogs. Breed characteristics and fur type could explain this observation, as well as variations in participants’ characteristics or needs. Overall, these results offer insight into the changes in engagement among participants, therapists, and dogs throughout DAT sessions.

The analysis of the focus direction indicates that most participants in this study initially focused on the dog, which may help ease them into the therapeutic process by providing emotional comfort (Coakley et al., 2020). Early research describes this as the “Icebreaker Effect” of the dog, making it easier for the therapist and participant to come into contact and create a safe atmosphere (Meixner & Kotrschal, 2022). Over time, the participants’ focus shifts toward the therapist, with a balance between the therapist, the dog, and the environment emerging by Session 5. By Session 9, the participant’s focus direction stabilises on the therapist, which fits with the dog’s role as a bridge to build trust, as A.IDEM Fine (2018) described. Joint focus patterns also show that participant-therapist engagement seems to increase in most cases, while participant–dog interaction slightly decreases. This transition highlights the evolving dynamic, where the dog facilitates early engagement, but the therapist becomes central for achieving therapeutic goals. However, this is not found for all participants. Interestingly, the focus between therapists and dogs remained stable, with only short moments of joint focus observed. This lack of joint focus between the therapist and the dog challenges assumptions about the dog–therapist dynamic (Mignot et al., 2022). At the same time, the therapist frequently observed the dog, with an average of 26% per session, indicating that the dog’s well-being was being monitored. This suggests that while there was limited collaboration between the therapist and the dog during the sessions, attention to the dog was present. Still, this raises questions about the exact nature and necessity of therapist–dog interaction as described by Glenk and Foltin (2021). Additionally, while the dogs’ well-being was safeguarded through recommended session durations, the lack of comparison with non-DAT sessions limits conclusions about DAT’s unique effects. Future studies should explore how different types of therapist–dog interaction might influence therapy.

It should be noted, however, that the therapist and the dog did observe each other more frequently than they jointly focused on a shared activity. This suggests that although there was limited joint focus—understood here as engaging together in a shared task—the therapist still monitored the dog, and the dog paid attention to the therapist. The distinction between joint focus and focus on each other is therefore not always clear-cut. Moreover, the less collaborative role of the therapist and dog during sessions may partially explain the low levels of joint engagement.

The literature suggests that DAT participants value its active nature and reduced focus on verbal interaction (Van Schooten et al., 2024). However, the results of our study indicate that participants could have been more active during the sessions, with participants actively participating only about one-third of the time, and this level of participation slightly declined from Session 1 to Session 9. Therapists demonstrated a similar trend, with their active involvement decreasing notably by Session 9. This could be attributed to the structured nature of the sessions, while one therapist had more experience, the others had less than two years of experience, which may have led to a closer adherence to the therapy protocol. This observation aligns with Aubrey Fine’s Handbook on animal-assisted therapy, which emphasises the importance of tailoring the structure of the sessions to meet therapeutic goals. The moderate to high levels of structure in this study may have limited unplanned interactions, while these unplanned, spontaneous interactions can encourage engagement between the participant and the dog. For future research, adopting a less rigid session structure to encourage more spontaneous participant–dog interactions could potentially provide richer data on the dynamics of DAT. Moreover, shorter observation intervals and a closer examination of the dog’s evolving role during different stages of therapy could yield additional insights into how these interactions contribute to the therapeutic process.

The analysis of physical contact between the participant and the dog revealed notable differences. While these data must be interpreted cautiously, examining the differences between these outcomes seems important. The literature shows that physical contact between participants and dogs can affect the neurobiological systems, influencing oxytocin levels, heart rate, and bonding behaviour (Beetz et al., 2012). These are critical factors that can shape the therapeutic process and contribute to building a strong alliance. Based on the literature, having physical contact appears beneficial for participants and dogs. Our data indicate that participants who worked with the Poodle had significantly more physical contact. Possible explanations include (1) the dog’s temperament and natural need for affection and (2) the dog’s coat type, as Poodles have softer, non-greasy, and non-shedding fur (Udvarhelyi-Tóth et al., 2024). Given the numerous positive effects of physical contact, further research is needed to investigate these interactions and determine whether physical contact correlates with other therapeutic outcomes. Dogs with more affectionate personalities or softer coats may naturally foster more participant engagement, potentially enhancing the therapeutic experience. Exploring how these factors impact participant–dog interaction will provide valuable insights into how dogs affect the therapeutic process and could help to create a guideline to match the dogs with participants to maximise therapeutic effectiveness.

In addition to the dog’s physical characteristics regarding contact, an alternative explanation may lie in the type of participant. Research by Nagasawa et al. (2015) demonstrates that women tend to have a stronger inclination for closeness with dogs, linked to oxytocin-mediated bonding. Notably, the two participants who interacted with the more “cuddly” dog in our study were both older adolescent girls. Their choice of this particular dog may reflect a greater need or preference for physical closeness and affectionate moments compared to the younger participants. This interplay between participant characteristics and dog selection could have influenced the observed frequency of physical contact. On the other hand, other research indicates that men may engage in more physical contact with unfamiliar dogs in therapeutic or structured settings, where the dog is not their own (Shih et al., 2020). Given these mixed findings, it remains unclear to what extent participant gender or preferences influence physical contact with dogs, highlighting the need for further research to clarify this relationship.

Two challenges of this study were the use of a low-frequency behaviour analysis approach and the heterogeneity of the participant group. The low-frequency behaviour analysis method provided a broad overview of the movement and focus direction towards the therapist, participant, and dog. It is inappropriate to capture brief yet meaningful interactions, such as short “check-in moments” between the therapist and dog (looking at each other) or short physical contact between the participant and the dog. The 30 s recording intervals excluded high-frequency behaviours and subtle nuances of the therapeutic process. Future research could address this limitation by implementing shorter intervals, such as 5 s, and focusing on key exercises or meaningful moments rather than entire sessions. This adjustment would allow for a more precise and detailed analysis of behaviours while managing data complexity effectively. The second challenge was the heterogeneity of the participant group. The wide age range (12 to 18 years) introduces developmental differences in maturity, attention span, and emotional regulation, potentially affecting consistency of engagement, which makes comparison and generalisation difficult. While all participants faced social–emotional or focus-related challenges, the specific nature of these issues differed, influencing how they interacted with the dog, therapist, and therapy process. This variability complicated the analysis of participation and focus patterns, as observed behaviours could reflect individual differences rather than therapy effects. To address this, future research should consider more narrowly defined inclusion criteria to create a more homogeneous group.

## 5. Conclusions

This study examined interactions in DAT sessions, revealing a general trend toward increased participant–therapist engagement over time, while participant–dog interaction tended to decrease. This partially supports the “icebreaker” theory, which suggests that the dog initially facilitates rapport between the participant and therapist, after which the therapist–participant relationship becomes more prominent. However, this trend was not consistent across all participants. Similarly, therapist–dog interactions remained low throughout and varied only minimally. These individual differences underline the importance of further investigating the dynamics.

## Figures and Tables

**Table 1:** The therapist focuses on the participant, the dog, and other aspects of each session.

Focus Direction	Towards Participant			Towards Dog			Other		
Session	1	5	9	1	5	9	1	5	9
Participant 1	51%	65%	44%	21%	12%	37%	28%	24%	20%
Participant 2	61%	70%	69%	27%	16%	10%	12%	14%	21%
Participant 3	41%	64%	51%	43%	24%	36%	16%	12%	13%
Participant 4	62%	67%	66%	20%	14%	12%	18%	19%	20%
Participant 5	47%	57%	54%	38%	34%	42%	15%	9%	5%
Average	53%	64%	57%	30%	20%	27%	18%	16%	16%

**Table 2:** The participant’s focus direction toward the therapist, dog, and other aspects varies for each participant and each session in percentage.

Focus Direction	Towards Therapist			Towards Dog			Other		
Session	1	5	9	1	5	9	1	5	9
Participant 1	13%	13%	24%	36%	20%	48%	51%	67%	28%
Participant 2	38%	41%	60%	43%	23%	16%	19%	36%	25%
Participant 3	47%	63%	28%	40%	33%	49%	13%	4%	23%
Participant 4	70%	63%	72%	21%	21%	19%	9%	16%	9%
Participant 5	50%	55%	42%	42%	33%	31%	8%	12%	27%
Average	44%	47%	45%	37%	26%	33%	20%	27%	23%

**Table 3:** The dog’s focus direction towards the therapist, participant, and other aspects varies for each participant and each session in percentage.

Focus Direction	Towards Therapist			Towards Participant			Other		
Session	1	5	9	1	5	9	1	5	9
Participant 1	11%	16%	5%	24%	17%	5%	65%	67%	91%
Participant 2	8%	2%	5%	26%	17%	10%	66%	81%	85%
Participant 3	21%	13%	8%	16%	43%	50%	63%	44%	42%
Participant 4	4%	11%	8%	27%	26%	17%	69%	63%	74%
Participant 5	15%	8%	3%	33%	37%	36%	52%	55%	61%
Average	12%	10%	6%	25%	28%	24%	63%	62%	71%

**Table 4:** Joint focus during therapy sessions for each participant and each session is displayed in percentages.

	Participant–Dog Dyad			Therapist–Dog Dyad			Participant–Therapist Dyad		
Session	1	5	9	1	5	9	1	5	9
Participant 1	15%	4%	5%	3%	1%	1%	7%	12%	22%
Participant 2	21%	14%	6%	0%	1%	2%	32%	37%	55%
Participant 3	13%	28%	28%	4%	3%	2%	33%	55%	26%
Participant 4	14%	12%	12%	0%	2%	1%	56%	61%	64%
Participant 5	24%	28%	22%	6%	0%	2%	44%	51%	40%
Average	18%	17%	15%	3%	2%	2%	34%	43%	41%

## Data Availability

The datasets generated for this study are available on request to the corresponding author.

## Abbreviations

The following abbreviations are used in this manuscript:

ADHD	Attention-Deficit Hyperactivity Disorder
ASD	Autism Spectrum Disorder
BSI	Behavioural Science Institute, Radboud University
DAT	Dog Assisted Therapy
ECSW	Ethics Committee of the Faculty of Social Sciences, Radboud University
IAHAIO	International Association of Human–Animal Interaction Organizations

## Appendix A

### Appendix A.1. Ethogram

Behavioural observations are essential for systematically assessing interactions between dogs, clients, and therapists during animal-assisted interventions. An ethogram, which includes clearly defined behavioural categories, is used to ensure reliability and objectivity. This study will analyse video recordings of therapy sessions by pausing the video every 30 s to record observed behaviours. This sequential recording method allows for structured and comparable data collection.

#### Operational Definitions

1. Dog Behaviours:
  ➢ Dog lying: The dog rests on the ground with its body wholly or partially in contact with the floor.
  ➢ Dog sitting: The dog is seated with its hindquarters on the floor and front legs extended.
  ➢ Dog standing: The dog is on all four legs without movement.
  ➢ Dog walking: The dog moves forward or changes position using all four legs.
  ➢ Attention client: The dog is visibly oriented towards the client, with its head or body directed towards them.
  ➢ Attention therapist: The dog is visibly oriented towards the therapist, with its head or body directed towards it.
  ➢ Attention other: The dog is visibly oriented towards another individual or object in the environment.
  ➢ Dog makes physical contact: The dog initiates direct physical contact with the client, therapist, or another individual (e.g., nudging, leaning, pawing).
  ➢ Other: Any behaviour the client displays that does not fit the above categories.
2. Client Behaviours:
  ➢ Client sitting: The client is seated on a chair, bench, or the floor.
  ➢ Client standing: The client is upright and stationary.
  ➢ Client walking: The client is moving from one location to another.
  ➢ Attention dog: The client is visibly oriented towards the dog, with eye contact or body direction.
  ➢ Attention therapist: The client is visibly oriented towards the therapist, with eye contact or body direction.
  ➢ Attention other: The client is oriented towards another individual or object in the environment.
  ➢ Petting dog: The client physically touches or stroking the dog with their hands.
  ➢ Other: Any behaviour the client displays that does not fit the above categories.
3. Therapist Behaviours:
  ➢ Therapist sitting: The therapist is seated on a chair, bench, or the floor.
  ➢ Therapist standing: The therapist is upright and stationary.
  ➢ Therapist walking: The therapist is moving from one location to another.
  ➢ Attention dog: The therapist is visibly oriented towards the dog, with eye contact or body direction.
  ➢ Attention client: The therapist is visibly oriented towards the client, with eye contact or body direction.
  ➢ Attention other: The therapist is oriented towards another individual or object in the environment.
  ➢ Petting dog: The therapist physically touches or stroking the dog with their hands.
  ➢ Other: Any behaviour the therapist displays that does not fit into the above categories.
